# Multi Locus Sequence Typing of *Chlamydia* Reveals an Association between *Chlamydia psittaci* Genotypes and Host Species

**DOI:** 10.1371/journal.pone.0014179

**Published:** 2010-12-02

**Authors:** Yvonne Pannekoek, Veerle Dickx, Delphine S. A. Beeckman, Keith A. Jolley, Wendy C. Keijzers, Evangelia Vretou, Martin C. J. Maiden, Daisy Vanrompay, Arie van der Ende

**Affiliations:** 1 Department of Medical Microbiology, Academic Medical Center, Center for Infection and Immunity Amsterdam (CINIMA), Amsterdam, The Netherlands; 2 Department of Molecular Biotechnology, Faculty of Bioscience Engineering, Ghent University, Ghent, Belgium; 3 Department of Zoology, University of Oxford, Oxford, United Kingdom; 4 Laboratory of Biotechnology, Department of Microbiology, Hellenic Pasteur Institute, Athens, Greece; Duke University Medical Center, United States of America

## Abstract

*Chlamydia* comprises a group of obligate intracellular bacterial parasites responsible for a variety of diseases in humans and animals, including several zoonoses. *Chlamydia trachomatis* causes diseases such as trachoma, urogenital infection and lymphogranuloma venereum with severe morbidity. *Chlamydia pneumoniae* is a common cause of community-acquired respiratory tract infections. *Chlamydia psittaci*, causing zoonotic pneumonia in humans, is usually hosted by birds, while *Chlamydia abortus*, causing abortion and fetal death in mammals, including humans, is mainly hosted by goats and sheep. We used multi-locus sequence typing to asses the population structure of *Chlamydia*. In total, 132 *Chlamydia* isolates were analyzed, including 60 *C. trachomatis*, 18 *C. pneumoniae*, 16 *C. abortus*, 34 *C. psittaci* and one of each of *C. pecorum*, *C. caviae*, *C. muridarum* and *C. felis*. Cluster analyses utilizing the Neighbour-Joining algorithm with the maximum composite likelihood model of concatenated sequences of 7 housekeeping fragments showed that *C. psittaci* 84/2334 isolated from a parrot grouped together with the *C. abortus* isolates from goats and sheep. Cluster analyses of the individual alleles showed that in all instances *C. psittaci* 84/2334 formed one group with *C. abortus*. Moving 84/2334 from the *C. psittaci* group to the *C. abortus* group resulted in a significant increase in the number of fixed differences and elimination of the number of shared mutations between *C. psittaci* and *C. abortus*. *C. psittaci* M56 from a muskrat branched separately from the main group of *C. psittaci* isolates. *C. psittaci* genotypes appeared to be associated with host species. The phylogentic tree of *C. psittaci* did not follow that of its host bird species, suggesting host species jumps. In conclusion, we report for the first time an association between *C. psittaci* genotypes with host species.

## Introduction

*Chlamydia* comprises a group of obligate intracellular bacterial parasites responsible for a variety of diseases in humans and animals, including several zoonoses. It was proposed in 1999 that the single genus of *Chlamydia* should be reassigned into two genera, *Chlamydia* and *Chlamydophila*, based on clustering analyses of the 16S rRNA and 23S rRNA genes [1], which has not been widely accepted by the chlamydial research community. Recently however, reversion to the single genus *Chlamydia* was recommended [2], with the *Chlamydia* nomenclature used here.

*Chlamydia trachomatis* can cause diseases with severe morbidity, such as trachoma, urogenital infection and lymphogranuloma venereum [3]–[5]. Several serovars and genotypes have been identified, but which have not been linked to disease or clinical outcome [6], [7]. *Chlamydia pneumoniae* is a common cause of community-acquired pneumonia, bronchitis, pharyngitis and sinusitis [8]. Although *C. pneumoniae* often causes mild or subclinical infections, its persistence in the host can lead to the establishment of chronic pathologies and has been implicated with arteriosclerosis [9] and coronary heart diseases [10], [11]. *Chlamydia psittaci* which can cause zoonotic pneumonia in humans are usually hosted by birds [12]–[16]. Transmission of *C. psittaci* from birds to humans is frequently reported and veterinarians, poultry farmers, bird breeders and pet shopkeepers are in particular at high risk [17]–[27].

*Chlamydia abortus* has been associated with abortion and fetal death in mammals, including humans, and is hosted by goats, sheep and less frequently by cattle, horses and pigs [28]. The microorganism has also been associated with pneumonia, conjunctivitis, arthritis as well as epididymitis and has been isolated from the faeces of healthy sheep and goats [29]–[32]. Pregnant women are at risk when exposed to animals infected with *C. abortus* and may suffer severe infections, including spontaneous abortion [33]–[36].

Multi-locus sequence typing (MLST) based on the partial sequences of seven housekeeping genes was first used to evaluate the population structure of *Neisseria meningitidis* [37]. Previously, we developed an MLST scheme to understand the population genetic structure of *C. trachomatis* and *C. pneumoniae* and the diversity of these species and to evaluate the association between genotype and disease [7]. In the present study, the MLST scheme was used to evaluate the population structure of *C. psittaci* and *C. abortus*. Results of cluster analyses of concatenated sequences of the 7 housekeeping fragments expanded and validated the proposed typing system for all chlamydial species. The results indicated that the 7 housekeeping fragments used in our study were likely representative for the whole genome sequence. *C. psittaci* genotypes were associated with their host species; the *C. psittaci* phylogenetic tree however, did not follow that of its host bird species. Furthermore, *C. psittaci* 84/2334, formerly considered as the missing link between *C. abortus* and *C. psittaci*, was clearly typed as *C. abortus*.

## Results

### Population structure of *Chlamydia*

**Figure 1:**
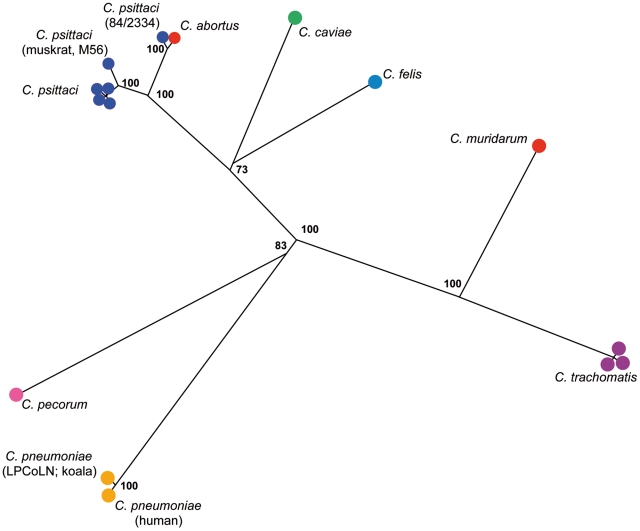
Phylogenetic analyses of concatenated sequences of 7 housekeeping gene fragments of *Chlamydia* strains. Concatenated sequences were aligned and analysed in MEGA 4.0.2. Phylogenetic tree was constructed using the Neighbour-Joining algorithm using Maximum Composite Likelihood model. Bootstrap test was for 1000 repetitions. Bold numbers indicate bootstrap values over 50% of the main branches.

Among 132 *Chlamydia* isolates 44 sequence types (STs) were identified; 19 STs among 60 *C. trachomatis* isolates, 4 STs among 18 *C. pneumoniae* isolates, 12 STs among 34 *C. psittaci* isolates and 4 among 16 *C. abortus* isolates (Table S1). Previously, it was shown that in *C. trachomatis* the homologous recombination rate is low [38]. We determined the index of association for the different *Chlamydia* species according to Haubold [39]. Significant linkage disequilibrium was detected for *C. psittaci*, *C. abortus*, *C. trachomatis* and *C. pneumoniae* (Table S2). In addition, we tested the sequences for evidence of recombination using the maximum chi square [40]. Recombination events were not detected in *C. pneumoniae* and *C. abortus* sequences. In *C. trachomatis*, 5 putative recombination events were detected between a pair of *hflX* alleles (putative recombination site at position 422) and 4 *oppA* alleles (putative recombination sites at positions 500 and 506), respectively. In *C. psittaci,* we detected one putative recombination event in *hemN* (at position 426) (Table S3). Together these data suggest that the role of recombination in diversity of *Chlamydia* species is low and that *Chlamydia* species are clonal. In phylogenetic analyses with clonal bacterial species, where mutation is more important than recombination, it is preferable to use concatenated sequences of the MLST loci rather than allelic profiles, because the magnitude of changes between alleles is lost in allelic profiles [41]. This method was subsequently used to study relatedness of closely related species [42], [43], [44]. Phylogenetic analysis of all 132 strains of *Chlamydia* using the Neighbour-Joining algorithm with the maximum composite likelihood model of the single 3120 base pairs (bp) sequence of the aligned concatenated loci resulted in a tree comparable to that obtained with 16S rRNA gene and 23S rRNA gene sequences [1] and to the previous reported tree based on concatenated sequences of 6 MLST loci [7] (Fig. 1). In the present tree, three main groups are identified among 60 *C. trachomatis* isolates, consistent with earlier reported results of analyses of 26 *C. trachomatis* isolates (not shown) [7]. In addition, *C. pneumoniae* LPCoLN isolated from koala branched separately from *C. pneumoniae* from patients, consistent with the results of Myers and colleagues [45]. *C. psittaci* M56 from a muskrat branched separately from the main cluster of *C. psittaci* isolates, while *C. psittaci* 84/2334 grouped together with *C. abortus*, suggesting that 84/2334 belongs to *C. abortus*.

### Diversity among *Chlamydia* species

Concatenated sequences of the MLST loci were used to estimate the diversity among and divergence between *Chlamydia* species. The diversity among *C. trachomatis* and *C. pneumoniae* is limited (Table 1). Nucleotide substitutions in protein encoding genes can be either synonymous or non-synonymous (resulting in a changed amino acid). Darwinian selection may lead to the retention of non-synonymous substitutions. The number of synonymous and non-synonymous substitutions may indicate the degree of selection operating in the population. The average number synonymous substitutions per synonymous site d_S_ in *C. trachomatis* and *C. pneumoniae* were comparable, while the average number of nonsynonymous substitutions per nonsynonymous site d_N_ was three times higher among *C. trachomatis* (Table 1). However, considering *C. pneumoniae* from humans only, than d_S_ in *C. pneumonia* was much lower than that in *C. trachomatis* and d_N_ of *C. pneumonia* even 7 fold lower than that of *C. trachomatis*, indicating that *C. pneumoniae* from human is much more clonal than *C. trachomatis* in accordance with previous observations [7].

**Table 1:** Diversity of *Chlamydia*.

species	number of isolates	number of bp sequenced	d_S_ *	number of synonymous substitutions		d_N_	number of nonsynonymous substitutions	d_N_/d_S_
*C. trachomatis*	60	3080	0.00226	11		0.00135	15	0.598
*C. pneumoniae*	18	3102	0.00224	12		0.00037	5	0.164
*C. pneumoniae* from humans only	17	3102	0.00076	2		0.00019	1	0.245
*C. psittaci* all	34	3096	0.02115	177		0.00151	38	0.072
*C. psittaci* excluding M56 and 84/2334	32	3096	0.00606	22		0.00058	7	0.095
*C. abortus* all	16	3096	0.00245	9		0.00020	2	0.080
*C. abortus* including *C. psittaci* 84/2334	17	3096	0.00382	16		0.00022	2	0.058

*d_S_ and d_N_: the average number of synonymous substitutions per synonymous site and nonsynonymous substitutions per nonsynonymous site, respectively (Jukes-Cantor corrected).

The d_S_ and d_N_ among the 34 *C. psittaci* isolates was much higher than among any other *Chlamydia* included in this study (Table 1). However, the d_S_ of *C. psittaci* without M56 and 84/2334 was considerably lower, but still higher than among *C. pneumoniae*, *C. abortus* or *C. trachomatis*. The d_S_ and d_N_ among the 16 *C. abortus* isolates were comparable to those among the *C. pneumoniae* isolates, reconfirming the homogeneity observed in this species, in accord with previous results [46]. Inclusion of *C. psittaci* 84/2334 to *C. abortus* increased the diversity among the species, which remained lower than the diversity among *C. psittaci* without M56 and 84/2334 (Table 1). The degree of selection can be expressed by the d_N_/d_S_ ratio; a ratio d_N_/d_S_ higher than 1 indicates a positive selection (altered amino acid substitutions are common), while a rate lower than 1 indicates negative selection (silent substitutions). The *C. trachomatis* and *C. pneumoniae* have the highest d_N_/d_S_, but in both species synonymous substitutions are in excess (Table 1).

### Divergence between *Chlamydia* species

The divergences between *Chlamydia* species adjacent in the tree can be expressed as the average number of nucleotide substitutions per site between two species populations (D_XY_) and the number of fixed differences, i.e. mutations uniform within the species populations. Since most fixed differences are neutral and accumulate at a rate proportional to the mutation rate (molecular clock), the number of fixed differences are indicative for the elapsed time since two populations evolved from a common ancestor. The D_XY_ between *Chlamydia* species closely positioned in the N-J tree were similar with two exceptions (Table 2). First, the divergence between *C. pneumoniae* and *C. pecorum* was twofold higher than that between other neighbours in the tree. Of note, the D_XY_ and the number of fixed differences between *C. pneumoniae* from human and LPCoLN was small and consistent with the results obtained by comparisons of available *C. pneumoniae* genome sequences by Myers and colleagues [45]. Second, the D_XY_ between *C. psittaci* and *C. abortus* was remarkably low and the number of fixed differences was even lower when compared to that between *C. pneumoniae* isolated from human and LPCoLN (Table 2). In addition, only *C. abortus* and *C. psittaci* shared polymorphisms. However, while the D_XY_ between *C. psittaci* without 84/2334 and *C. abortus* with 84/2334 was similar to that between the whole *C. psittaci* and *C. abortus* populations, the number of fixed differences was considerably higher, but still much lower than between other neighbouring species in the tree. In addition, shared polymorphism were absent between *C. psittaci* without 84/2334 and *C. abortus* with 84/2334. The divergence between 84/2334 and *C. abortus* was limited and of the same magnitude as that between human *C. pneumoniae* and LPCoLN, supporting the notion that 84/2334 and *C. abortus* are one species.

**Table 2:** Divergence between *Chlamydia* species.

species	D_xy_ *	No. of fixed differences (no. of shared polymorphism)
*C. trachomatis* vs *C. muridarum*	0.19362	521 (0)
*C. pneumoniae* vs *C. pecorum*	0.38094	915 (0)
*C. pneumoniae* from human vs LPCoLN from koala	0.00474	14 (0)
*C. felis* vs *C. caviae*	0.18023	496 (0)
*C. abortus* including 84/2334 vs *C. caviae*	0.18160	491 (0)
*C. abortus* including 84/2334 vs *C. felis*	0.19958	533 (0)
*C. psittaci* all vs *C. abortus* all	0.05677	3 (3)
*C. psittaci* excluding 84/2334 vs *C. abortus* including 84/2334	0.05833	132 (0)
*C. psittaci* excluding M56 and 84/2334 vs M56	0.02066	53 (0)
*C. abortus* vs 84/2334	0.00362	7 (0)

*D_XY_: Average number of nucleotide substitutions per site between populations (Jukes-Cantor corrected).

### *C. psittaci* 84/2334 belongs to *C. abortus*

**Figure 2:**
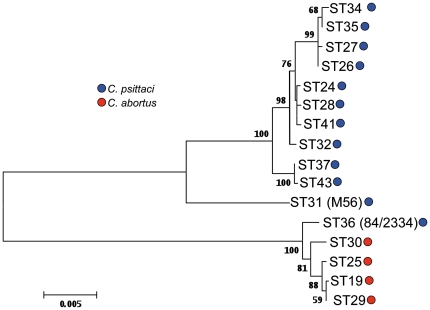
Phylogenetic analyses of concatenated sequences of 7 housekeeping gene fragments of *C. psittaci* and *C. abortus* strains. Concatenated sequences were aligned and analysed in MEGA 4.0.2. Phylogenetic tree was constructed using the Neighbour-Joining algorithm using Maximum Composite Likelihood model. Bootstrap test was for 5000 repetitions. Numbers indicate bootstrap values over 50%. Only unique genotypes (STs) were included in the clustering analyses. STs are displayed in Table S1.

The N-J tree clearly showed that *C. psittaci* M56 branched separately from the main group of *C. psittaci* strains isolated mainly from birds, while *C. psittaci* 84/2334 grouped together with *C. abortus* isolates (Fig. 1 and 2). Cluster analyses of the 7 individual housekeeping gene fragments showed that with four gene fragments M56 grouped close to the remaining (except 84/2334) *C. psittaci* isolates. With gene fragments *gatA*, *hflX* and *fumC* M56 grouped neither with *C. psittaci* nor with *C. abortus*. Cluster analyses of the 7 individual housekeeping gene fragments showed that in all instances 84/2334 grouped together with *C. abortus* (Fig. S1). In addition, the *hemN* sequence of 84/2334 was identical to that of all but one *C. abortus* isolates. Together, with the analyses of the divergence between *C. abortus* and *C. psittaci* (Table 2) these results suggest that 84/2334 belongs to *C. abortus*.

### *C. psittaci* genotypes associate with host species

Using BURST ST24 and ST28 were defined as singletons, although 13 isolates from parrots/parakeets were ST24 (group I) and 8 isolates (7 from ducks and one from human) were ST28 (group II). Two clonal complexes or groups were defined: group III comprises 5 isolates from pigeons and one isolate from human and group IV was formed by the two isolates from turkey (data not shown).

**Figure 3:**
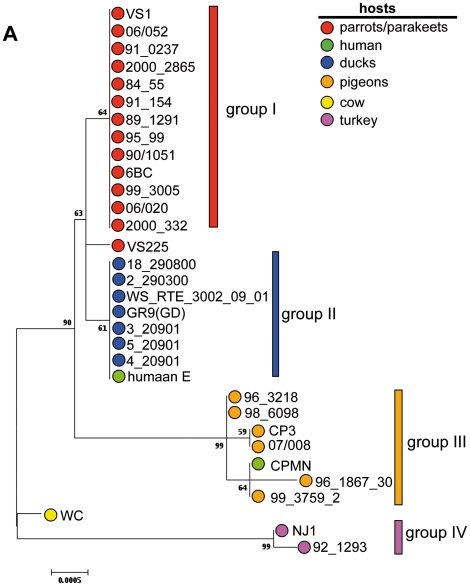
Phylogenetic analyses of concatenated sequences of 7 housekeeping gene fragments of *C. psittaci* strains, excluding strain 84/2334 and M56. Concatenated sequences were aligned and analysed in MEGA 4.0.2. Phylogenetic tree was constructed using the Neighbour-Joining algorithm using Maximum Composite Likelihood model. Bootstrap test was for 5000 repetitions. Numbers indicate bootstrap values over 50%.

Results of cluster analyses performed with concatenated sequences of MLST loci of *C. psittaci* excluding 84/2334 and M56 showed association between host species and *C. psittaci* genotype with the aforementioned four groups being recognized (Fig. 3). One isolate, *C. psittaci* VS225 from a parakeet, was found to lie between the group I isolates from parrots/parakeets and the group II isolates from duck. The *C. psittaci* WC bovine isolate did not group with any of the four main groups. Clustering was not associated with the geographic origin of the isolates or of their corresponding host species as parrots/parakeets which have their origin in three different continents, grouped together (Table S1).

## Discussion

In a previous study we used the present MLST scheme to analyze clonal groupings among *C. trachomatis* and *C. pneumoniae* strains [7]. A database hosted at http://pubmlst.org/chlamydiales/was developed and sited at the University of Oxford. The website offers a large number of ways to query the database and to further break down and export the results [47]. In the present study we used the MLST scheme to explore the population genetics of *C. psittaci* and *C. abortus* isolates. The main findings are that *C. psittaci* genotypes are associated with their host species and that isolate 84/2334, formerly classified as *C. psittaci*, is most likely typed as *C. abortus.*

We have shown in our previous study that an UPGMA tree produced from the allelic profiles and from concatenated allele sequences of 28 *C. trachomatis* isolates resulted in three groups of sequence types [7]. The urogenital strains were distributed over two separated groups; one consisted solely of strains with the frequent occurring serovars E, D and F. Strains isolated from patients with lymphogranuloma venereum (LGV strains) grouped in a single cluster, which also included *C. trachomatis* B/TW5, although not being identical. The close relatedness of B/TW5 was supported by results of comparisons of IncA sequences, showing that B/TW5 shares IncA polymorphisms with LGV strains, which were not found among other serovars [6]. The present study, with *C. trachomatis* strains supplemented with isolates from a study by Ikryannikova and colleagues [48] to a total of 60, yielded the same three groups in cluster analyses. Recently, another MLST scheme was described showing clonal groupings among *C. trachomatis* strains with a group consisting exclusively of LGV strains [49]. These results differ somewhat from those reported in our previous study [7].

Recently, a phylogenetic tree was reported that was based on concatenated sequences of 110 conserved protein sequences extracted from the available whole genome sequences of *C. trachomatis*, *C. pneumoniae*, *C. pecorum*, *C. felis*, *C. psittaci*, *C. abortus* and *C. caviae* [2], [45]. Our tree that is based on the concatenated sequences of 7 housekeeping fragments is very similar to the original tree. In addition, we showed that in our tree *C. pneumoniae* LPCoLN isolated from koala branched separately from the remaining human *C. pneumoniae* strains consistent with the results reported by Myers and colleagues who compared whole genome sequences of LPCoLN and *C. pneumoniae* AR39, TW-183, CWL029 and J138 isolated from human providing evidence that humans were originally infected zoonotically [45]. Together, these results indicate that the 7 housekeeping fragments used in our study are representative for the whole genome sequence.

Phylogenetic analyses of the concatenated allele sequences of *C. psittaci* revealed an association between *C. psittaci* genotype and host species. Genotype was not associated with geographic origin, as the host birds had their natural habitat in different countries and covering four different continents, yet the *C. psittaci* isolated from them were of a single genotype. This identified association between *C. psittaci* genotype and host species has not been previously observed, which may due to the difference in sequences studied. Cluster analyses of *C. psittaci* has previously been performed using *ompA* [50], [51], *ompA* based PCR-RFLP [52] or multi loci variable number of tandem repeats (MLVA) [53]. *ompA* encodes the major outer membrane protein and is subjected to host immuno-pressure and its sequence may therefore not reflect the genetic make up of *Chlamydia*. MLVA based on genetic variation of tandem repeats may be also less suitable to assess evolutionary history by phylogenetic analyses, because it lacks sequence information.

Recently Hackett and colleagues examined ∼32 kilobases of aligned nuclear DNA sequences from 19 independent loci for 169 bird species, representing all major extant groups, and recovered a robust phylogeny from a genome-wide signal supported by multiple analytical methods [54]. In their phylogenetic tree based on Maximum Likelhood analyses ducks and turkeys group relatively close together, while pigeons and parrots are more distantly related. In contrast, our results show that *C. psittaci* from ducks grouped closer to those from parrots and more distantly from those isolated from turkeys, indicating that *C. psittaci* phylogeny did not follow that of their host species birds. Possibly, *C. psittaci* may have been transferred from Psittaciformes (order of birds to which parrots and parakeets belong) to Ansiformes (order of birds to which ducks belong) or vice versa. Alternatively, *C. psittaci* has been transferred to different bird species more than once from a source other than birds. Both scenarios imply host species jump. Incidental transmission is not likely because *C. psittaci* isolates from these birds and from ducks were isolated in different countries and as far as known in different years. In addition, *C. psittaci* M56 from a muskrat has it own branch in the phylogenetic tree obtained by using concatenated sequences of MLST loci similar as in a split network graph based on a sequence similarity matrix of *ompA* sequences reported by Sachse and colleagues [51]. This and the observation, that *C. psittaci* WC from a cow also does not group with *C. psittaci* from birds, might indicate that *C. psittaci* may have spread among mammals, allowing for the development of mammal species specific *C. psittaci* genotypes as well. Analyses of more *C. psittaci* isolates from different host species will shed more light on this interesting question. Recently Mitchell and colleagues showed that *C. pneumoniae* found among animals is more divers than those found among humans [55]. In addition, their results support two separate animal-to-human cross species transfer events in the evolutionary history of this pathogen. Although *C. psittaci* has a broad host range and numerous cases of transmissions from birds to humans have been described [16], reports of human to human transmission of *C. psittaci* are rare [21], [56], indicating that *C. psittaci* has not yet adapted to the human host.

Of note, two *C. psittaci* strains were isolated from human, suggesting that these were incidental zoonotic infections, without establishing *C. psittaci* in humans. *C. psittaci* CPMN isolated from human and thereafter repeatedly passaged in ferrets [57] and *C. psittaci* humaan E strain grouped together with strains isolated from pigeons and from ducks, respectively. Interestingly, the latter case was a turkey farmer, of whom pharyngeal and nasal swab were taken before the arrival of a new flock of turkeys arrived at the farm and repeatedly thereafter [24]. Initially, *C. psittaci* with *ompA* genotype E was cultured, but after three to six weeks *ompA* genotype A was found, the same *ompA* genotype found among the turkeys. Our data suggest that the initial infection may be acquired from waterfowl.

The 16 *C. abortus* isolates comprised 4 STs (5 when 84/2334 is included). Of note, strains LLG and POS have the same genotype and grouped separately from the remaining *C. abortus* isolates, consistent with the results of Laroucau and colleagues using MLVA [46] and immunological analysis [58]. Furthermore, MLST analyses in this study differentiated the vaccine strain 1B from its parental strain AB7, both classified as the same genotype by MLVA [46]. The 4 *C. abortus* genotypes were not grouped according to their host species (sheep or goat).

The much lower divergence between *C. psittaci* and *C. abortus* compared to that between the other species, suggests *C. psittaci* and *C. abortus* have diverged from a common ancestor and much more recently than the others in the tree have from their common ancestor. Alternatively, *C. psittaci* might have been transmitted from birds to ruminants and adapted to the new hosts as already suggested by Pudjiatmoko and colleagues [59]. In our study, host species jump by *C. abortus* may be indicated by *C. psittaci* 84/2334, which grouped close to all *C. abortus* strains, showing limited divergence with *C. abortus* with only 7 fixed nucleotide substitutions and should therefore be classified as *C. abortus*. Originally, *C. psittaci* 84/2334 was isolated from a yellow-crown amazon (parrot) and classified as *C. psittaci* based on its reaction with specific sera against the major outer membrane protein (MOMP). However, among 60 *C. psittaci* isolates from birds, strain 84/2334 had a unique *ompA AluI* restriction pattern, indicating that this strain differs from the majority of *C. psittaci* strains [60]. It was suggested that *C.psittaci* 84/2334 was an intermediate between *C. psittaci* and *C. abortus* based on analysis of *ompA*, *rnpB* and the *rrn* (part of the region between the 16S rRNA 23SrRNA genes) sequences [50]. In addition, *C.psittaci* 84/2334 was found to have DNA sequences that were identical to an extrachromosomal plasmid in duck *C. psittaci* strain N352, while extrachromosomal plasmids are not found in *C. abortus* strains [50]. However, trees based on full length 16S rRNA and 23S rRNA gene sequences showed that *C. abortus* strains grouped together with *C. psittaci* strains [1]. Similar results were obtained with cluster analyses based on 390 bp *rnp* sequences. In an N-J tree based on these sequences *C. psittaci* 6BC and *C. abortus* B577 were indistinguishable [61]. Cluster analyses of *rnp* sequences extracted from GenBank showed *C. psittaci* 84/2334 grouped closer to *C. abortus* isolates than to *C. psittaci* isolates (data not shown). Also, the difference in the 222 base pairs *rrn* sequence between *C. psittaci* strains and *C. abortus* strains is limited. *C. psittaci* 84/2334 differs at only one position with 5 *C. abortus* isolates of which their *rrn* sequences are publicly available. *C. psittaci* 84/2334 and the 5 *C. abortus* isolates differ at 3 to 4 positions with *C. psittaci* strains. Again, in a tree based on *rrn* sequences extracted from GenBank, *C. psittaci* 84/2334 grouped closer to *C. abortus* than to *C. psittaci* (data not shown). In addition, an extended *rrn* sequence of 315 bp of *C. psittaci* 84/2334 also differed at only one position from the *C. abortus* sequences [62]. In our cluster analyses we used up to 10-fold more sequences information than the single 16S RNA, 26S rRNA, *rnn* and *rnp* sequences and showed clear separation between *C. psittaci* isolates and *C. abortus* isolates with one exception: *C. psittaci* 84/2334 grouped together with *C. abortus*. Cluster analyses of the individual housekeeping fragments showed that in all instances strain 84/2334 grouped together with *C. abortus*. Nevertheless, for six of the seven housekeeping fragments strain 84/2334 has a unique allele. This could indicate that strain 84/2334 is unique within the group of *C. abortus* isolates. In addition, only *C. abortus* from a limited set of host (goats and sheep) have been included in the analyses. Inclusion of more *C. abortus* isolates from different hosts will most likely result in more *C. abortus* genotypes some of these maybe more related or identical to that of *C. psittaci* 84/2334. Ultimately, whole genome sequences of more *C. psittaci* strains isolated from different host including *C. psittaci* 84/2334 will be ideal to assess these questions.

In conclusion, *C. psittaci* genotypes are associated with host species and *C. psittaci* 84/2334 should be reclassified as C. *abortus*.

## Methods

### Strains

All 132 *Chlamydia* isolates (currently present in the MLST data base for *Chlamydiales* at http://pubmlst.org/chlamydiales/) were included in the study (Supplementary Table S1). This includes 26 *C. trachomatis* and 16 *C. pneumoniae* strains from a earlier study [7] and 30 *C. trachomatis* strains submitted to the MLST database by Dr. Ikryannikova, who kindly gave her permission to use the data [48]. Detailed analyses were performed with 34 *C. psittaci* strains isolated from different bird species and mammals from different geographic locations and 16 *C. abortus* strains. For comparison, sequences of *C. caviae*, *C. felis*, *C. pecorum* and *C. muridarum* were also included [7]. *C. psittaci* strains were isolated form dead animals brought to the veterinary clinic for autopsy, or from samples sent to the laboratory by veterinary practitioners for chlamydial diagnosis. *C*. *abortus* B577 (VR656) was purchased from ATCC. Greek *C. abortus* strains were isolated from infected placentae or aborted fetuses. *C. abortus* MA/231184, MB/312, ME/4004, MF/337, MD/3920, FAS, FAG, VPIG and the variant strains LLG and POS have been previously described, as well as the reference strains AB7, A22, and S26/3 [58], [46]. The vaccine strain 1B (Enzovax) was purchased from Intervet (Intervet-Hellas) and strain Krauss-15 was kindly provided by Dr Jones (Moredun Research Institute, UK). *C. psittaci* was cultured in Buffalo green monkey cells (ATCC CCL-26) identifying the organisms by the IMAGEN™ *Chlamydia* immunofluorescence staining (Dakocytomation, Denmark), as previously described [24] and *C. abortus* stocks were produced in McCoy cell monolayers in the continuous presence of cycloheximide (1 µg/ml) and subsequently typed with monoclonal antibodies against the major outer membrane protein (MOMP) and the polymorphic membrane proteins (Pmps) as previously described [58].

### DNA, genes, PCR products and sequences

DNA extraction, PCRs and DNA sequencing were performed as previously described [7]. All alleles of the partial sequences of the 7 housekeeping genes are accessible via the MLST website for *Chlamydiales* (http://pubmlst.org/chlamydiales/). New allele sequences have been deposited in GenBank (accession no.: HM776459-HM776511).

### Phylogenetic and other analyses

Sequences of fragments from seven housekeeping genes (*enoA*, *fumC*, *gatA*, *gidA*, *hemN*, *hlfX*, *oppA*) were analyzed as previously described [7]. Allele numbers and genotypes were identified at http://pubmlst.org/chlamydiales/. Clonal complexes were identified using BURST in the START2.0 software package at http://pubmlst.org/software/analysis/start2/ [63]. Clonal complexes consisted of sequence types that shared 6 of 7 alleles with at least 1 other sequence type in the complex. The index of association for the different *Chlamydia* species according to Haubold [39] and sequences were tested for recombination using maximum chi square [40] with the START2.0 software package.The number of synonymous and non-synonymous substitutions per site was determined using DnaSP 4.0 [64]. Phylogenetic and molecular evolutionary analyses of the seven housekeeping fragments (individually or concatenated) were conducted using MEGA version 4 [65] or SplitsTree version 4.0 [66], generating a Neighbor-Joining (N–J) tree using the Maximum Composite Likelihood model.

## Supporting Information

Table S1 All isolates used in this study according to *Chlamydia* species, host and ST.
(0.12 MB XLS)

Table S2 Detecting linkage disequilibrium in MLST data of *Chlamydia* species.
(0.03 MB DOC)

Table S3 Detection of putative recombination events in *Chlamydia* species.
(0.04 MB DOC)
