# Supplementary material for: Multi Locus Sequence Typing of Chlamydia Reveals an Association between Chlamydia psittaci Genotypes and Host Species
Source: PLoS One. 2010 Dec 2;5(12):e14179. doi: 10.1371/journal.pone.0014179 (PMC2996290; doi:10.1371/journal.pone.0014179)
Supplement: Table S2 — Detecting linkage disequilibrium in MLST data of Chlamydia species. (0.03 MB DOC) [file pone.0014179.s002.doc]

| **Species** | **V_o_** | **V_e_** | **I_a_** | **Max trial variance** | **L_mc_** | **P** | **Significant linkage disequilibrium detected** |
| --- | --- | --- | --- | --- | --- | --- | --- |
| *C. trachomatis* | 3.7115 | 1.4626 | 0.2563 | 1.7985 | 1.6414 | <0.0001 | yes |
| *C. pneumoniae* | 2.4412 | 0.7906 | 0.3479 | 1.5596 | 1.0859 | <0.0001 | yes |
| *C. psittaci* all | 6.239 | 1.469 | 0.5412 | 2.2568 | 1.8282 | <0.0001 | yes |
| *C. psittaci* excluding M56 and 84/2334 | 5.7878 | 1.4393 | 0.5035 | 2.3252 | 1.7312 | <0.0001 | yes |
| *C. abortus* all | 4.3529 | 1.1003 | 0.4927 | 1.5596 | 1.6806 | <0.0001 | yes |
| *C. abortus* including *C. psittaci* 84/2334 | 5.9102 | 1.351 | 0.5624 | 2.5041 | 2.0732 | <0.0001 | yes |

V_o_: observed mismatch variance, V_e_: expected mismatch variance, I_a_: index of association, L_mc_: simulated 5% critical value.
