# Supplementary material for: Multi Locus Sequence Typing of Chlamydia Reveals an Association between Chlamydia psittaci Genotypes and Host Species
Source: PLoS One. 2010 Dec 2;5(12):e14179. doi: 10.1371/journal.pone.0014179 (PMC2996290; doi:10.1371/journal.pone.0014179)
Supplement: Table S3 — Detection of putative recombination events in Chlamydia species. (0.04 MB DOC) [file pone.0014179.s003.doc]

| **Spcecies** | **Sequence 1** | **Sequence 2** | **Putative recombination site** | **Max χ^2^** | ***P*** |
| --- | --- | --- | --- | --- | --- |
| *C. trachomatis* | hflX-3 | hflX-4 | 422 | 26.4354 | 0.07 |
|  | oppA-5 | oppA-6 | 500 | 100.3937 | 0.013 |
|  | oppA-3 | oppA-6 | 506 | 126.7485 | 0.021 |
|  | oppA-4 | oppA-6 | 506 | 62.499 | 0.037 |
|  | oppA-1 | oppA-6 | 506 | 41.0856 | 0.049 |
| *C. psittaci* | hemN-9 | hemN-11 | 426 | 71.1647 | 0.021 |
|  | hemN-11 | hemN-13 | 426 | 34.6675 | 0.049 |
| *C. psittaci* excluding M56 and 84/2334 | hemN-9 | hemN-11 | 426 | 71.1647 | 0.03 |
| *C. abortus* | none |  |  |  |  |
| *C. abortus* including *C. psittaci* 84/2334 | none |  |  |  |  |
| *C. pneumoniae* | none |  |  |  |  |
